# Artificial Intelligence’s Performance on the Japanese National Dental Examination

**DOI:** 10.7759/cureus.73103

**Published:** 2024-11-05

**Authors:** Tatsuya Akitomo, Masakazu Hamada, Yasuko Tsuge, Ami Kaneki, Masashi Ogawa, Taku Nishimura, Satoru Kusaka, Chieko Mitsuhata, Ryota Nomura

**Affiliations:** 1 Department of Pediatric Dentistry, Graduate School of Biomedical and Health Sciences, Hiroshima University, Hiroshima, JPN; 2 Department of Oral and Maxillofacial Oncology and Surgery, Graduate School of Dentistry, The University of Osaka, Osaka, JPN; 3 Department of Pediatric Dentistry, Hiroshima University Hospital, Hiroshima, JPN

**Keywords:** artificial intelligence, chatgpt, dental education, japanese national dental examination, learning

## Abstract

Background/purpose: Artificial intelligence (AI) has developed rapidly and is applied to many fields including dental education. In this study, we evaluated AI performance on the Japanese National Dental Examination.

Materials and methods: We extracted 349 of 400 compulsory questions from the National Dental Examinations over the past five years. Questions were presented to ChatGPT 3.5, ChatGPT 4o mini, and Gemini, and their performance was evaluated across 13 topic categories.

Results: ChatGPT 4o mini achieved the passing criteria for exams for two years and had the highest total score of the three AIs. The scores of ChatGPT 4o mini on “Society and dentistry” and “Cardinal signs” were significantly higher than those of ChatGPT 3.5 (*P*<0.05).

Conclusions: The high performance of ChatGPT 4o mini indicates the potential value of the tool in dental education. Further improving its performance may lead to future clinical applications in dentistry.

## Introduction

Modern generative artificial intelligence (AI) models typified by Stable Diffusion (generating images) and GPT3 (generating text) are among the most exciting developments in AI research [1]. In recent years, the AI language generation model has been applied to chatbots such as ChatGPT [1]. ChatGPT’s remarkable capabilities stem from this two-part approach: leveraging GPT3’s modeling of human language while fine-tuning it to generate new text that is conversational and responsive to human feedback [1].

Reports suggest that ChatGPT can pass a number of higher education and professional board exams, achieving more than 50% accuracy across all three U.S. Medical Licensing Examinations and an average of over 60% [2]. Kawahara et al. (2024) investigated AI performance on the Japanese National Medical Licensing Examination and reported that the AI exceeded the minimum score required by examinees over each of the past six years [3]. These results suggest high AI performance in medical examinations and great utility in the medical education field.

Japanese National Dental Examination consists of required basic topics, general dentistry, and each topic of dentistry [4]. The required basic items are called “compulsory questions.” They are considered to constitute the basic knowledge and skills necessary to become a dentist. It consists of 13 basic categories [4,5]. AI performance on the Japanese National Dental Examination has been investigated [4]. However, there is no report on the performance of AI models regarding the 13 basic categories of compulsory questions. In addition, ChatGPT 4o mini, the revised version of ChatGPT, was released in July 2024 and its usefulness is currently unknown. Thus, in this study we investigated the performance of three types of AI, including ChatGPT 4o mini, on the Japanese National Dental Examination.

## Materials and methods

Ethical approval was not required because this study investigated the usefulness of AI. We extracted the compulsory questions of the National Dental Examination over the past five years from 2020 to 2024. The questions were obtained from the Ministry of Health, Labor and Welfare website [6], yielding a total of 349 questions from the total of 400 questions, excluding questions that were difficult to reproduce by text because they contained diagrams and charts. Additionally, questions that every AI could not answer at the time of our study were also excluded. In July 2024, the questions were presented to the free version of ChatGPT 3.5 (OpenAI Global, San Francisco, California, U.S.) and Gemini (Google, Mountain View, California, U.S.). In addition, ChatGPT 4o mini (OpenAI Global, San Francisco, California, U.S.), the revised free version, was used in September 2024. Scores were calculated as follows: (number of correct answers) / (number of questions: 349) × 100. The pass mark was set at 80% in line with the national examination. In addition, the questions were classified into 13 topics against which the AI performance was evaluated. Statistical analyses were conducted using GraphPad Prism 9 (GraphPad Software Inc., La Jolla, CA, USA). Student’s t-test was used to test between two groups, and differences were considered statistically significant at *P*<0.05.

## Results

Scores of each AI by year

The compulsory questions cover 13 basic categories (Table 1).

**Table 1:** 13 basic categories of the Japanese National Dental Examination

Basic categories
1. Medical ethics and dental professionalism
2. Society and dentistry
3. Team medical care
4. Prevention and health management/promotion
5. Normal structure and function of the human body
6. Human body development, growth, and aging
7. Etiology and pathogenesis of major diseases and disorders
8. Cardinal signs
9. Basics of medical examination
10. Basics of examination and clinical diagnosis
11. Emergency care
12. Fundamentals of treatment and basic techniques
13. General education

**Figure 1:**
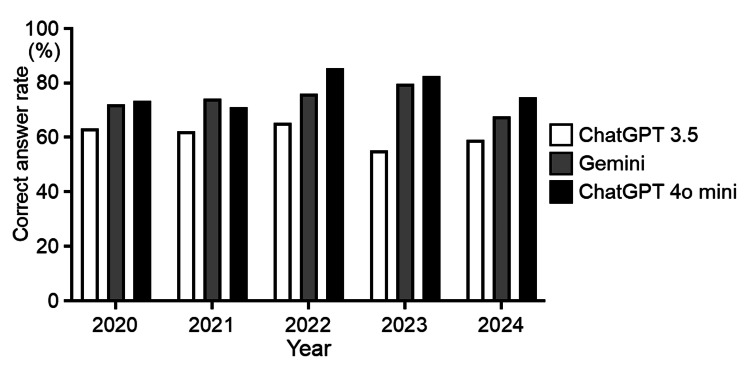
The AI scores for the national dental examination ChatGPT 3.5: OpenAI Global, San Francisco, California, U.S.; Gemini: Google, Mountain View, California, U.S.; ChatGPT 4o mini: OpenAI Global, San Francisco, California, U.S.

AI scores from 2020 to 2024 are shown in Figure 1.

ChatGPT 3.5’s scores ranged from 55.1% to 65.3%, and those of Gemini ranged from 67.6% to 79.7%. In contrast, ChatGPT 4o mini's scores ranged from 71.2% to 85.3%, which obtained the highest score for four years, except for 2021, and achieved the passing criteria in two years.

Scores of each AI for 13 basic categories

The comparison of performance across the 13 topics is shown in Table 2.

**Table 2:** Mean scores of AI for 13 basic categories of the Japanese National Dental Examination Student’s *t*-test was used to test between two groups, ^*^P<0.05, ^**^P<0.01, ^***^P<0.001 versus Gemini; ^†^*P*<0.05, ^†††^*P*<0.001 versus ChatGPT 4o mini. ChatGPT 3.5: OpenAI Global, San Francisco, California, U.S.; Gemini: Google, Mountain View, California, U.S.; ChatGPT 4o mini: OpenAI Global, San Francisco, California, U.S.

	Mean score (%)		
Basic categories	ChatGPT 3.5	Gemini	ChatGPT 4o mini
1	63.6	81.8	90.9
2	58.7^†^	73.9	80.4
3	66.7	73.3	73.3
4	64.1	66.7	74.4
5	64.9	70.3	83.8
6	55.0	60.0	70.0
7	63.2	71.1	73.7
8	42.9^†^	61.9	76.2
9	63.2	73.7	73.7
10	76.5	88.2	94.1
11	61.3^** †^	93.5	87.1
12	54.8^*^	83.9	64.5
13	100.0	100.0	100.0
Total	61.0^***, †††^	73.9	77.7

ChatGPT 4o mini had the highest score in 11 categories; its scores on “Society and dentistry” and “Cardinal signs” were significantly higher than those of ChatGPT 3.5 (*P*<0.05). In the “Emergency care” category, ChatGPT 3.5 showed significantly lower scores ​​than the other AI (*P*<0.05). Gemini’s score was the highest in “Fundamentals of treatment and basic techniques” and differed significantly from that of ChatGPT 3.5. All the AIs responded correctly to the “General education” question. The total score of ChatGPT 4o mini was the highest, showing a significant improvement from the previous version (*P*<0.001).

## Discussion

In this study, we assessed AI performance on the Japanese National Dental Examination, and ChatGPT 4o mini achieved a score worthy of passing. Although it has been reported that AI can achieve more than 70% on national medical examinations (although not on questions with images) [3], scores in the present study ranged from 61.0 to 77.7%. According to the Ministry of Health, Labor and Welfare website, the pass rate for the National Medical Examination over the past five years has exceeded 90%. However, the pass rate for the National Dental Examination has been in the 60% range [6]. The characteristics of the National Dental Examination may be associated with the AI scores.

We classified the questions into 13 topics that revealed AI’s strengths and weaknesses. ChatGPT can provide general and basic-level medical information. However, more details and specific treatment options still need to be obtained through a medical professional [7]. In the present study, AI ​​showed high scores in areas requiring a single answer, such as “General Education.” However, recent National Dental Examinations have become more difficult, requiring candidates to diagnose illnesses and select treatment plans based on symptoms. Further advances in AI are required to answer complex questions.

The questions used in this study were basic ones from the National Dental Examination, and questions including images are expected to be more difficult. In fact, Kawahara et al. (2024) showed that scores for questions including images were about 5% lower than other questions [3]. The present study was conducted using a free version of AI that is accessible to everyone. Additional verification of the findings, including questions that contain images, is needed in the future.

Previous analysis of ChatGPT and Gemini has been undertaken in a health sciences education context [8]. Ranjan et al. (2024) investigated the performance of ChatGPT 3.5 and Gemini on 200 microbiology questions, demonstrating comparable accuracy with correct response scores [9]. In the present study, Gemini showed a higher accuracy rate than ChatGPT 3.5. However, the latest version, ChatGPT 4o mini, had the highest score among the three AI types. The strength and nature of AI vary across AI tools, making it important to understand each one’s characteristics when selecting them for use.

This study has a limitation. In this study, ChatGPT 3.5 and Gemini were investigated at the same time; however, the evaluation period of ChatGPT 4o mini was different because it was released afterward. The results of this study may vary depending on the time of the survey; however, re-examination may also affect the results because AI is developing every day. Conducting the investigation at a standardized evaluation period allows for more accurate comparisons, and in addition, continuing to evaluate regularly leads to clearly the development of AI in the future.

## Conclusions

In this study, we used the free version of AI; on the other hand, the paid version can handle images. Further investigation between the free version and the paid version is needed. However, ChatGPT 4o mini, a revised free version of ChatGPT, met the passing requirement of the Japanese National Dental Examination, excluding questions that included images. In addition, it showed the highest score in 11 categories of 13 basic topics among three AIs, and the total score of ChatGPT 4o mini was significantly higher than that of ChatGPT 3.5 (*P*<0.001). These results show the rapid development being made in AI and suggest its potential for clinical application in the dental field.

## References

[1] Oermann EK, Kondziolka D (2023). On chatbots and generative artificial intelligence. Neurosurgery.

[2] Tam W, Huynh T, Tang A, Luong S, Khatri Y, Zhou W (2023). Nursing education in the age of artificial intelligence powered Chatbots (AI-Chatbots): are we ready yet?. Nurse Educ Today.

[3] Kawahara T, Sumi Y (2024). GPT-4/4V's performance on the Japanese National Medical Licensing Examination. Med Teach.

[4] Morishita M, Fukuda H, Muraoka K (2024). Evaluating GPT-4V's performance in the Japanese national dental examination: a challenge explored. J Dent Sci.

[5] (2024). Blueprint (National Dentist Examination Design Table). https://www.mhlw.go.jp/content/10803000/000920683.pdf.

[6] (2024). The Ministry of Health, Labor and Welfare of Japan. https://www.mhlw.go.jp/index.html.

[7] Ruksakulpiwat S, Kumar A, Ajibade A (2023). Using ChatGPT in medical research: current status and future directions. J Multidiscip Healthc.

[8] Shukla R, Mishra AK, Banerjee N, Verma A (2024). The Comparison of ChatGPT 3.5, Microsoft Bing, and Google Gemini for diagnosing cases of neuro-ophthalmology. Cureus.

[9] Ranjan J, Ahmad A, Subudhi M, Kumar A (2024). Assessment of artificial intelligence platforms with regard to medical microbiology knowledge: an analysis of ChatGPT and Gemini. Cureus.

